# Finite Element Modeling of Multilayer Orthogonal Auxetic Composites under Low-Velocity Impact

**DOI:** 10.3390/ma10080908

**Published:** 2017-08-05

**Authors:** Lili Jiang, Hong Hu

**Affiliations:** Institute of Textile and Clothing, Hong Kong Polytechnic University, Hung Hom, Kowloon, Hong Kong; lili.jiang@connect.polyu.hk

**Keywords:** finite element modeling, multilayer orthogonal auxetic composites, low-velocity impact, negative Poisson’s ratio

## Abstract

The multilayer orthogonal auxetic composites have been previously developed and tested to prove that they own excellent energy absorption and impact protection characteristics in a specific strain range under low-velocity impact. In this study, a three dimensional finite element (FE) model in ANSYS LS-DYNA was established to simulate the mechanical behavior of auxetic composites under low-velocity drop-weight impact. The simulation results including the Poisson’s ratio versus compressive strain curves and the contact stress versus compressive strain curves were compared with those in the experiments. The clear deformation pictures of the FE models have provided a simple and effective way for investigating the damage mechanism and optimizing the material, as well as structure design.

## 1. Introduction

### 1.1. Auxetic Composites

The auxetic materials, namely the materials with a negative Poisson’s ratio (NPR), have been studied for over three decades. Since the first auxetic polyurethane (PU) foam with a re-entrant structure was made by Lakes in 1987, a number of auxetic materials have been proposed and fabricated, ranging from macroscopic and micro-structural levels to a molecular level, including auxetic polymeric foams and micro-porous polymers [1,2,3,4,5,6,7], auxetic fibers [8,9] and fabrics [10,11,12,13], auxetic honeycombs [14] and composites [15,16,17,18], and so on. Auxetic materials have aroused much interest due to their counterintuitive deformation behavior and improved mechanical properties, such as enhanced shear resistance [19,20,21], increased indentation resistance [22,23,24,25], and improved crashworthiness [26,27]. These feature advantages have made auxetic materials very attractive for many potential applications such as automobile, aerospace and defense, and sport equipments, etc. [28,29], where impact protection can be one of the highly required properties.

The multilayer orthogonal auxetic composites with excellent impact resistance have been designed and fabricated in our previous work [30,31]. Compared with most commonly found auxetic materials, namely angle-ply laminates with auxetic effect and auxetic polymeric foams, the multilayer orthogonal auxetic composites have the features of large nonlinear deformation and remarkable structural integrity. Hence, they could be used individually or served as filling materials in smart structural components under different loading conditions, especially the multiple impact situation. They could find applications in sporting mats and the lining of protective helmets, etc. However, more experimental and numerical approaches should be adopted to fully explore the relationship between the macro mechanical properties of auxetic composites and the micro-structure design, as well as material selection.

Due to the limitation of the manufacturing cost and experimental limit, it is difficult to extend all the research work solely by experimental approaches. Finite element (FE) modeling methods are increasingly used to predict the mechanical response of composites and study the effects of various parameters. In this study, a three dimensional (3D) FE model in the ANSYS LS-DYNA package was established and verified to facilitate the parameters optimization of auxetic composites in the future.

### 1.2. FE Modeling of Impact on Foam-Filled Composites

The FE models on the dynamic impact properties of foams and foam-cored composites have been studied by many researchers [32,33,34,35,36,37,38,39,40,41,42,43,44]. Santosa and Wierzbicki [32,33] introduced 3D FE models on the crush behavior of aluminum foam-filled sections undergoing axial compressive loading. The effect of key geometrical parameters and the compressive resistance of foam on the mechanical properties of the foam-filled columns was investigated. A 3D FE model [34] in ANSYS LS-DYNA Explicit was built to analyze the dynamic response of a helmet-head form system under impact. The mechanical behavior of the composite shell and foam liner was modeled to reveal the mechanism of damage and failure of the composites.

Two dimensional (2D) random models for aluminum foam sandwich (AFS) panels with different relative densities were also created in ANSYS LS-DYNA [35]. The impact compression behavior and strain-rate effects of AFS panels were investigated based on the established FEM. It was concluded that the strain-rate effect of AFS panels was related to the strain rate sensitivity of matrix materials and effect of micro-inertia of AFS panels and the strain-rate effect of AFS panels became more obvious with the increase in relative density. Another 2D FE model [36] based on the Voronoi technique was carried out to investigate the energy absorption efficiency of density graded aluminum foam. The effects of the blast load impact velocity, loading duration, and sample thickness on the input energy density and output energy density of the graded foam were investigated. A parametric study showed that the density graded aluminum foam was effective in improving the energy absorption capability while maintaining a lower stress that was transmitted to the substrate or the protected objects if it was well designed.

Polymer foams, due to their large volumetric deformation characteristics, are difficult to model in FE analysis. There are several hyperelastic material models that have been developed for predicting the behavior of solid elastomers. In ABAQUS, the hyperfoam model is one of the most commonly used material models for predicting the behavior of polymer foam. The hyperfoam model can also be combined with linear viscoelasticity to create a rate dependent model [37]. In reference [38], a numerical model in ABAQUS was developed for open-cell flexible PU foam using the Ogden Hyperfoam material model. This model was implemented in simulating the Indentation Force Deflection test of the PU foam. A good correlation was found between the test and simulation results. The Ogden Hyperfoam material model was also used in reference [39] for the remoulded PU foam when the foam was used as head form crash mats for sports activities. The response of the PU foam was measured during the impact tests. The results showed that the FEA calculated head form forces were underestimated by about 35% compared with the results from the experimental drop tests due to the limitations in modelling a viscoelastic material with air compression contribution. In LS-DYNA, the flexible and open-cell PU foams suit the material model MAT57 well. The model incorporates both the loading and unloading behavior of materials. The parameters like the viscous coefficient and shape unload factor and hysteretic factor could be adjusted to control the stress-strain behavior of foams as indicated in the LS-DYNA Theory Manual [40,41,42].

FE modeling on the multiple compressive loading and unloading of expanded polystyrene foam was built in the paper [43,44]. The low density foam material model (MAT 57) in LS-DYNA accurately predicted the maximum deceleration, force, and displacement for first loading. However, for the case of unloading and reloading, the mechanical response and residual deformation of foam would be improved if the parameters for controlling shape and hysteresis in MAT 57 were calibrated using test results. In this study, the material model MAT57 in LS-DYNA was adopted to simulate the flexible PU foam in the FE model.

LS-DYNA is an advanced general-purpose FE simulation software package developed by the Livermore Software Technology Corporation (LSTC, Livermore, CA, USA) and it is capable of simulating complex nonlinear dynamic problems by using explicit time integration. The software has been widely used in the fields of automobile, aerospace, construction, military, manufacturing, and bioengineering [45]. ANSYS/LS-DYNA is a cooperating product between the LSTC and ANSYS Company (Pittsburgh, PA, USA), which is used to simulate the response of materials under various loading. The software is the most commonly used explicit FE simulation program and is very suitable for experienced and highly technical users. Many different types of elements, contact formulations, material models, and other controls in LS-DYNA code can be used to simulate the complex models with detailed problems [46].

## 2. FE Modeling of Auxetic Composite

### 2.1. Composite Structure and Parameters

**Figure 1:**
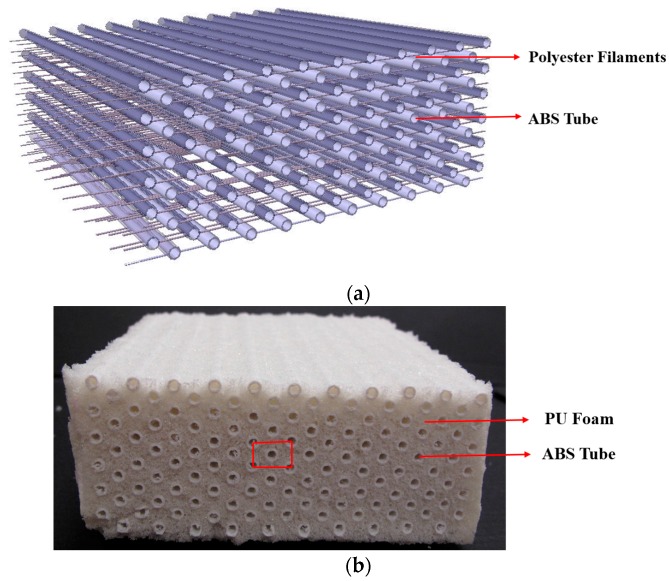
(**a**) The structure of the auxetic reinforcement; (**b**) The sample of the auxetic composite (the repeating unit is marked in the red frame).

The auxetic composites were fabricated via injecting and foaming techniques by using a multilayer auxetic orthogonal structure as the reinforcement and flexible open-cell PU foams as the matrix. The structure of the reinforcement is the same as used in reference [30], as shown in Figure 1a. Each auxetic composite, as shown in Figure 1b, is composed of three parts, which are the round acrylonitrile butadiene styrene (ABS) tubes (12 layers ABS tube thickness to decrease the boundary effect), the polyester filaments, and the matrix foam. The specifications of the polyester filaments and ABS tubes are relisted in Table 1. For the matrix material, flexible rebounded PU foam was utilized here to ensure better stress transmission and to increase the mechanical performance of auxetic composites after low velocity impact. The formulation of PU foam can be found in Table 2 of reference [47].

The outer diameter and wall thickness of the ABS tube are 3 and 0.4 mm, respectively. For the polyester filaments, their density and linear density are 1.38 g/cm^3^ and 167 tex. Suppose the cross-section of yarns is round, then their diameter (*d*) could be calculated from the yarn linear density (*Nt*) and density (*ρ*) according to Equation (1), which is 0.39 mm. However, in the real composite samples, the yarns were compressed to a flattened form, so that their cross sections were idealized as a slim rectangle of 0.6 mm × 0.2 mm to maintain the same cross section area of the circle state.

(1) $$d=3.5682\times 10^{-5}\times \sqrt{Nt/\rho }$$

*d* (unit: m), *Nt* (unit: tex), *ρ* (unit: g/cm^3^).

### 2.2. Experimental Testing and FE Geometric Modeling

**Figure 2:**
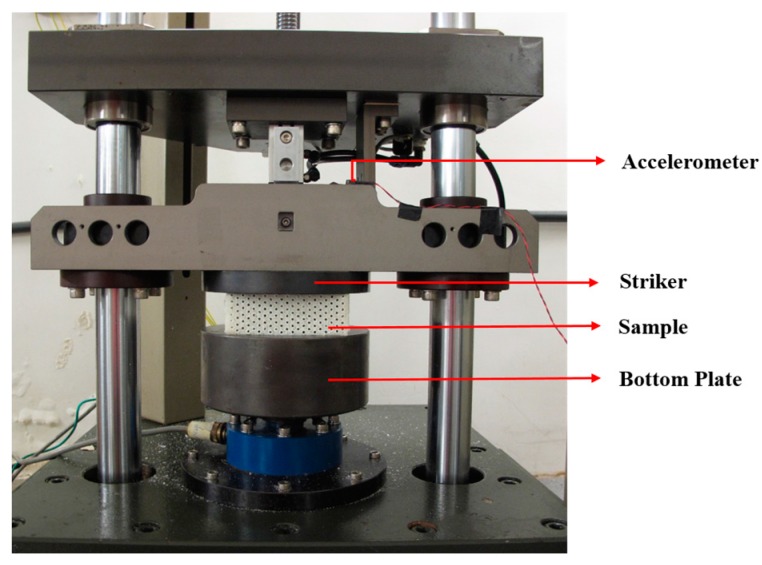
The drop-weight low-velocity impact testing system.

**Figure 3:**
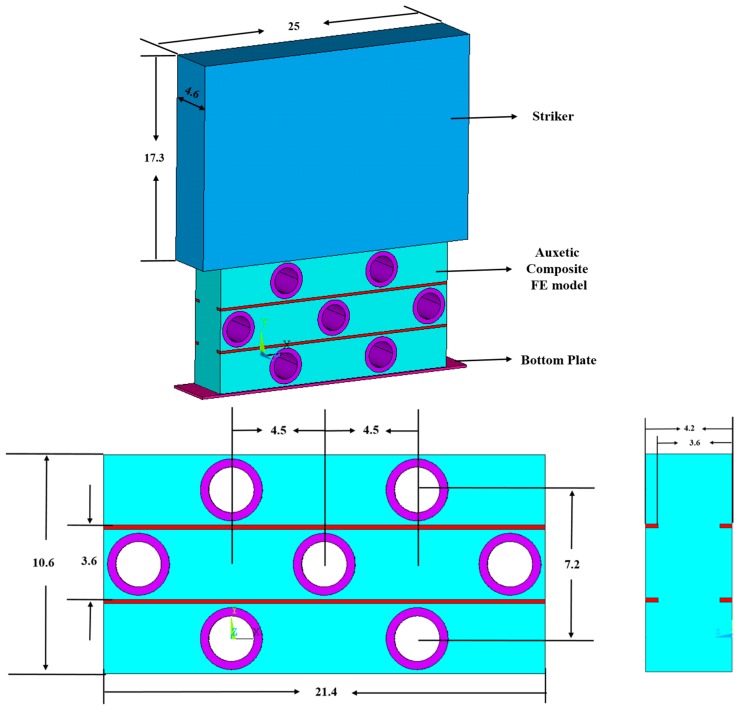
The geometry of the 3D FE model for the auxetic composite (unit: mm).

As shown in Figure 2, the low-velocity impact compression tests were conducted on a drop-weight impact testing system. During the test, the striker is released and hits the upper surface of the sample placed on the bottom plate. The striker weighs 6.5 kg and its striking surface is formed with a circular top plate of 150 mm in diameter. In the FE model, the striker is modeled as a hexahedron with a weight proportional to the actual striker in the experiment by having the same ratio between the actual size of the composite samples and the size of the FE model. As shown in Figure 3, the modeled striker has the dimension of 25 mm × 4.6 mm × 17.3 mm (width × thickness × height). This dimension was calculated from the fact that the percentage of the modeled striker volume divided by the actual impact striker is the same as that of the FE model volume divided by the actual sample. The striker is made of stainless steel. Its density is 7.8 g/cm^3^ and its weight is 6.5 kg. The dimensions of the FE model and the actual sample for impact testing are 21.4 mm × 10.6 mm × 4.2 mm and 98.0 mm × 97.6 mm × 41.8 mm, respectively. Moreover, the size of the striker surface is a little bigger than the dimension of the sample surface to ensure that the striker could fully cover and make contact with the testing samples. This was also considered in the calculation of the modeled striker’s dimension.

The element types for the ABS tubes, polyester filaments, and PU foams were set as SOLID 164, which is a type of element commonly used for the 3D modeling of solid materials [48]. The element is defined by eight nodes having the following degrees of freedom at each node: translations, velocities, and accelerations in the nodal x, y, and z directions. SOLID164 by default uses reduced (one point) integration and viscous hourglass control for faster element formulation. Due to the small size of the yarn cross section, the element meshes should be set to be small enough to ensure the calculation precision. If the mesh size is selected as 0.4 mm, the element number for the whole composite sample size will exceed two million. To save time and costs, a 3D FE model, which consisted of only two repeating units of an auxetic composite structure and could represent the impact behavior of the composite, was established in ANSYS/LS-DYNA, as shown in Figure 3. Seven ABS tubes and two polyester yarns were included in this model. The geometrical details of the model are also illustrated in Figure 3.

### 2.3. Element and Mesh Size

**Figure 4:**
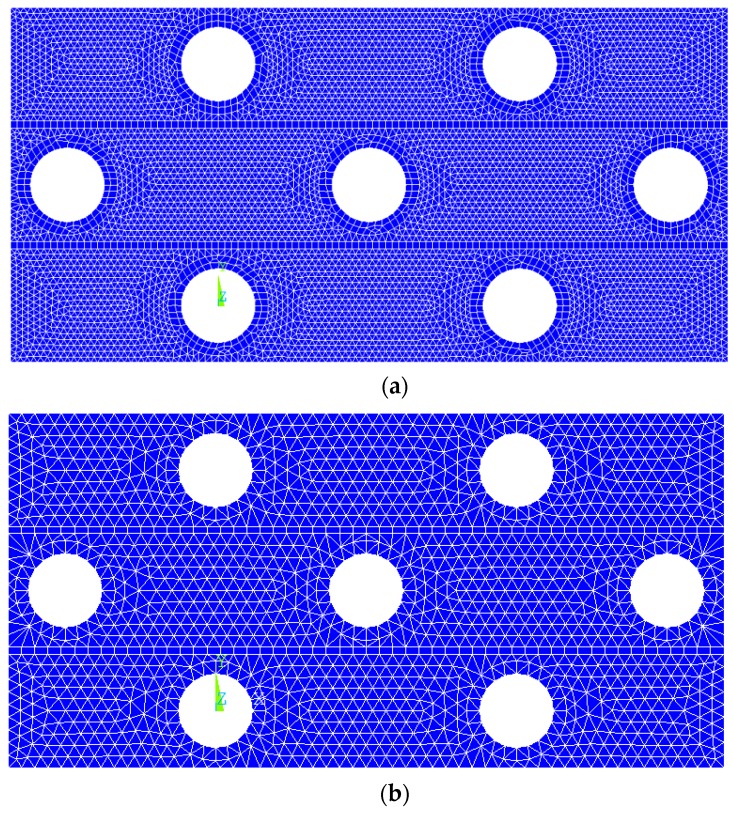
The FE models with different-size meshes: (**a**) element length = 0.2 mm; (**b**) element length = 0.4 mm.

**Figure 5:**
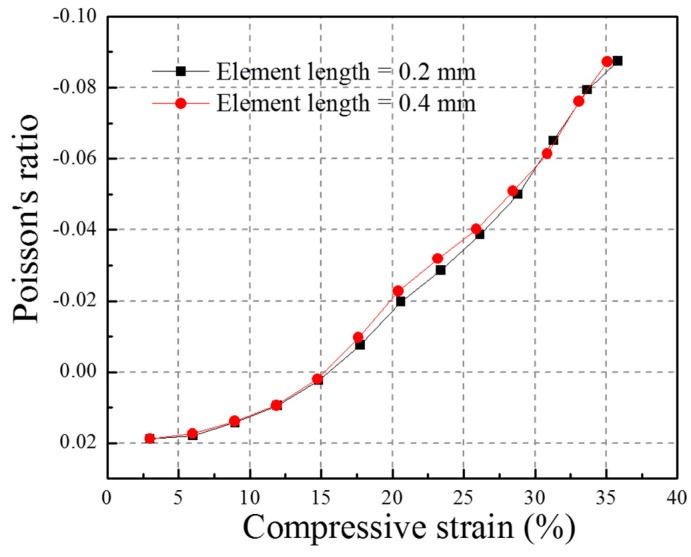
The Poisson’s ratio versus compressive strain curves for FE models with different element sizes (0.2 mm and 0.4 mm).

**Figure 6:**
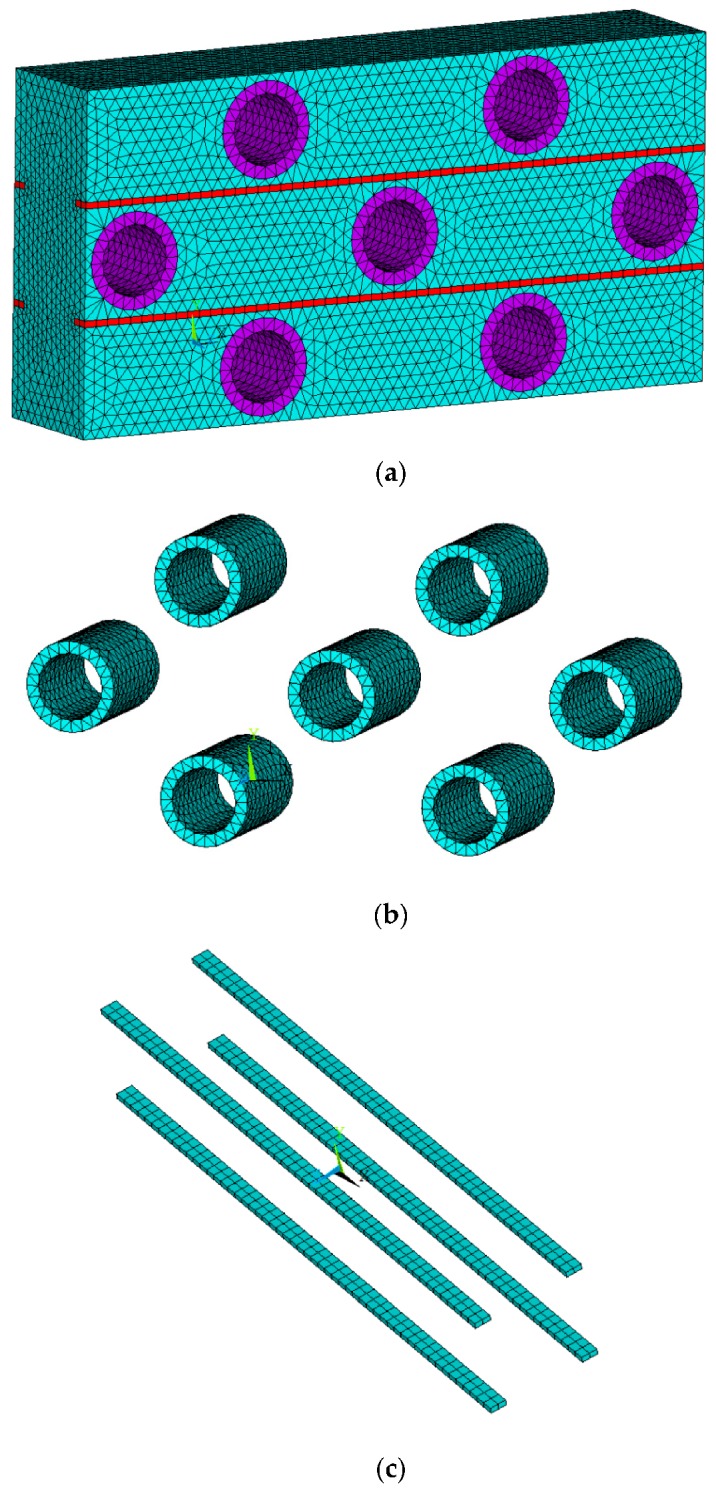
The FE models: (**a**) composite; (**b**) ABS tubes; (**c**) polyester yarns.

To determine the right size of mesh, the element lengths 0.2 mm and 0.4 mm were selected to mesh the FE model and their results on Poisson’s ratio versus compressive strain curves under the impact of 2.67 m/s were compared. The FE models with different-size meshes including 0.2 mm and 0.4 mm are shown in Figure 4. The corresponding Poisson’s ratio-compressive strain curves are presented in Figure 5 and the two Poisson’s ratio curves exhibit minor differences. The maximum NPR value for the FE models with a 0.2 mm and 0.4 mm mesh size are −0.08746 at the compressive strain of 35.81% and −0.08722 at the compressive strain of 35.08%, respectively. The difference between the two maximum NPR values is 0.28%, which is in an acceptable scope. Hence, the mesh size of 0.4 mm is sufficient for FE modelling. The FE models including the whole composite, ABS tubes, and polyester yarns are shown in Figure 6. The elements used for the matrix and tubes are tetrahedral elements and for yarns are brick elements. The total element number of the FE model is 113374.

### 2.4. Material Modeling

**Figure 7:**
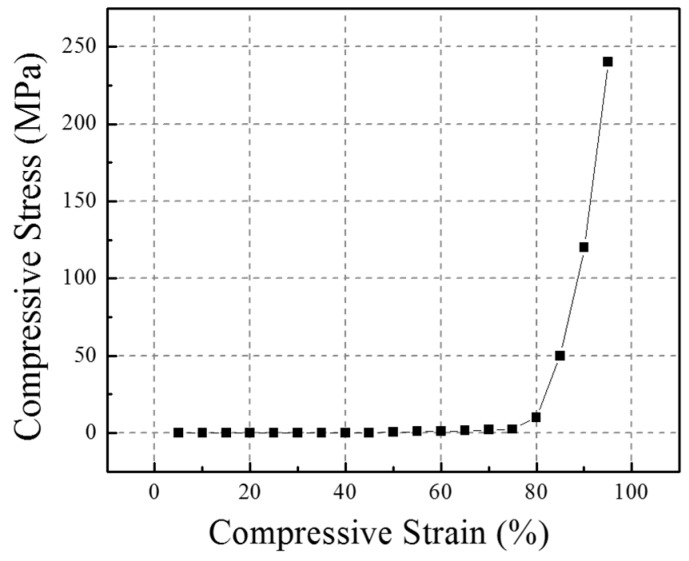
The extended compressive stress-strain curve of foam in FE analysis.

The material for the matrix of the composite is low density open cell PU foam. In LS-DYNA code, the MAT57 (Low Density Foam Material) [41,42] best suits the flexible PU foam in the FE model. It should be noted that the large deformation of foam elements could easily cause the negative volume error [49]. To avoid this error, the stress-strain curve of the PU foam was extended exponentially at large strains [50], as shown in Figure 7.

The detailed parameters for the foam material were as follows: Young’s modulus = 0.018 MPa, Density = 7.8 × 10^−11^ ton/mm^3^, Poisson’s ratio = 0, Tensile cutoff stress = 0.55 MPa, Viscous coefficient = 0.5, Hysteresis unload factor = 0.5, Shape unload factor = 0.5, Decay constant = 0, Failure option = 0, and Bulk viscous flag = 0. The stress-strain data was obtained from the curve, as shown in Figure 7, and they were then input into parameter tables embedded in ANSYS LS-DYNA. The hourglass control was set for the foam material. The hourglass type is 6 and the hourglass coefficient = 0.5.

**Figure 8:**
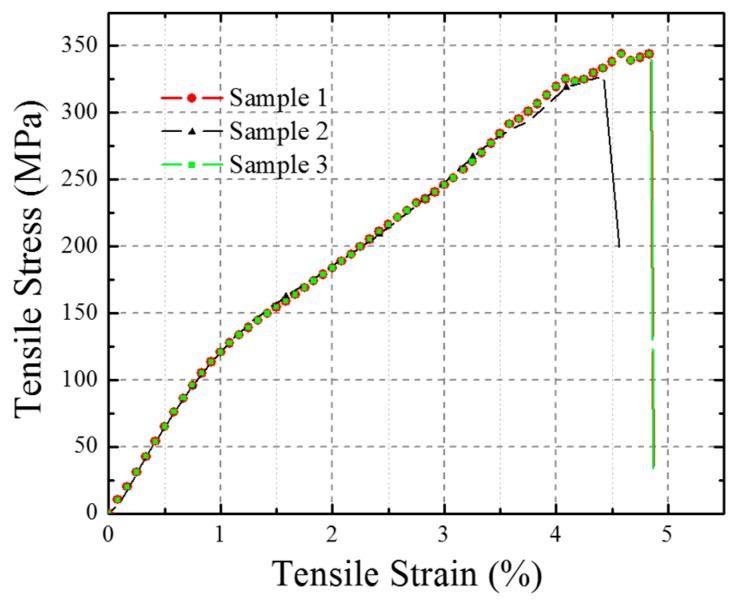
The tensile stress-strain curves of polyester filaments (three testing results).

The tensile stress-strain curve of the polyester filaments was obtained by a direct tensile test. As shown in Figure 8, the curve was split into three stages: the elastic stage, yielding region, and failure stage. Since the yarns in the composite only suffered during the elastic stage, the material properties for the polyester yarn in the FE model were assumed to be elastic and isotropic. The parameters are the Young’s modulus = 1.277 × 10^4^ MPa, Poisson’s ratio = 0.30, and Density = 1.38 × 10^−9^ ton/mm^3^.

The nature of the ABS plastic is firstly elastic and then plastic. Under impact, the ABS tubes in the composite mainly bear the elastic deformation, hence its material properties were set to be elastic and isotropic. The parameters are the Young’s modulus = 2.2 × 10^3^ MPa, Poisson’s ratio = 0.39, and Density = 1.05 × 10^−9^ ton/mm^3^.

The bottom plate and striker were made of rigid stainless steel, so their material properties were set to be rigid, linear, and isotropic: Young’s modulus = 2.07 × 10^5^ MPa, Poisson’s ratio = 0.30, and Density = 7.80 × 10^−9^ ton/mm^3^.

### 2.5. Contact and Constraints

The contact between the foam and yarns, foam, and tubes were idealized to be bounded. In others words, these three different materials were tied without any sliding. The contact between the striker and foam was set to be automatic surface to surface contact with a dynamic friction coefficient of 0.3.

For the boundary conditions, the bottom plate was fixed in all the directions of freedom and the bottom surface of the composite was also set to be DOF = 0. The mesh size of the striker and bottom plate was 1 mm.

Three different initial velocities of the striker were set as 1.50 m/s, 2.05 m/s, and 2.67 m/s.

## 3. Results and Discussion

The FE modeling was calculated and post-processed in the ANSYS LS-DYNA on the workstation with 16 CPUs and 16 GPa processors. The mechanical responses including the Poisson’s ratio versus compressive strain curves and the contact stress versus compressive strain curves were extracted and analyzed.

### 3.1. Deformation Process and Auxetic Effect

Due to the limitation of the impact testing conditions, it is hard to record the real deformation process of the samples. The FE method provides an easier and clearer way to observe and analyze the deformation and stress distribution of each component. This is meaningful for the investigation of the damage mechanism and the optimum design of the materials.

**Figure 9:**
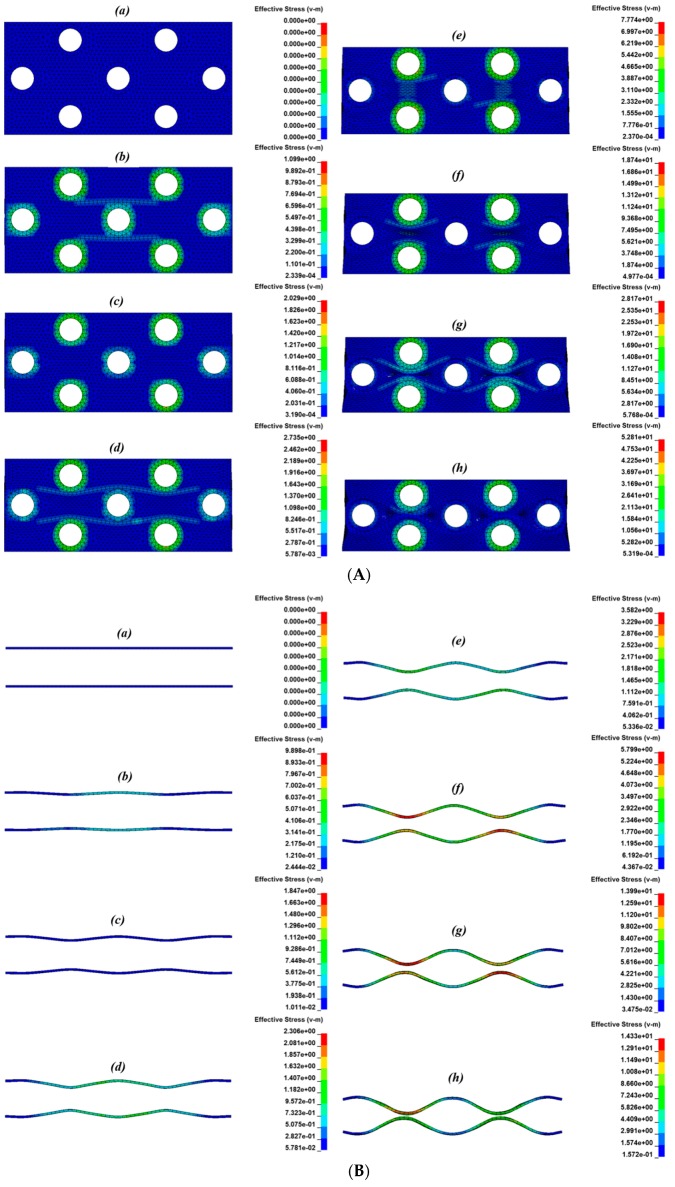

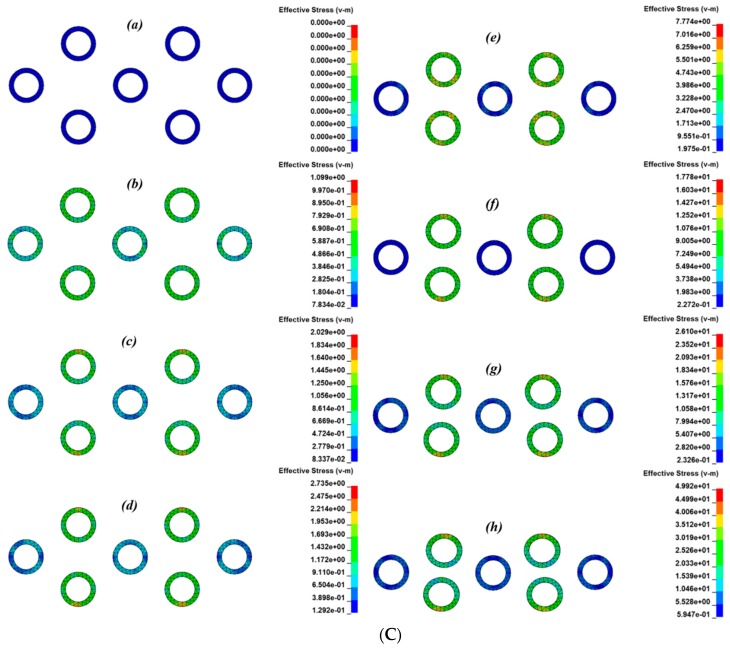
The impact deformation process and stress distribution of the auxetic composite at different compressive strains under an impact velocity of 2.67 m/s: (**a**) 0, (**b**) 5.60%, (**c**) 11.86%, (**d**) 17.61%, (**e**) 23.20%, (**f**) 28.45%, (**g**) 33.09%, (**h**) 35.08%. (**A**) Auxetic composite; (**B**) Yarns in composite; (**C**) Tubes in composite.

The deformation process and stress distribution of the auxetic composite, including the polyester yarns and ABS tubes in the composite at different compressive strains under an impact velocity of 2.67 m/s, are presented in Figure 9A–C. As illustrated in Figure 9A, under the impact of the striker, the composite gradually shrank in the horizontal direction from step (a) to step (h), which meant that the composite possessed a negative Poisson’s ratio in this direction and became denser to better resist the impact load. The mechanism of achieving an auxetic effect originates from the structure arrangement of the reinforcement and synchronistic effect of each constituent material in the composite. The structure of the auxetic composite in this study is a multilayered orthogonal structure and the component materials are rigid ABS open tubes, high-strength low-extension polyester yarns, and flexible open cell PU foam. During impact, the porous foam filling in other constituents firstly deformed and densified before the ABS tubes made contact with the polyester yarns. Then, the polyester yarns were slowly waved but not extended under the compression of ABS tubes due to their low bending stiffness and high elastic modulus (shown in Figure 9B). Meanwhile, the ABS tubes moved closer and closer due to the bending of yarns and shearing of PU foam, till the tubes lined up in the vertical direction and bore most of the compressive load. Finally, the composite began to reach the densified state. It can be seen from Figure 9C that the cross-sectional shape of the ABS tubes remained almost unchanged previous to step (g). From step (g) to (h), the ABS tubes became slightly flattened under the compression of the striker, which to some extent, decreased the auxetic effect of the composite. Step (h) was the last state of the composite under the impact process, at which point it then rebound step by step to the original state.

### 3.2. Poisson’s Ratio Versus Compressive Strain Curves

**Figure 10:**
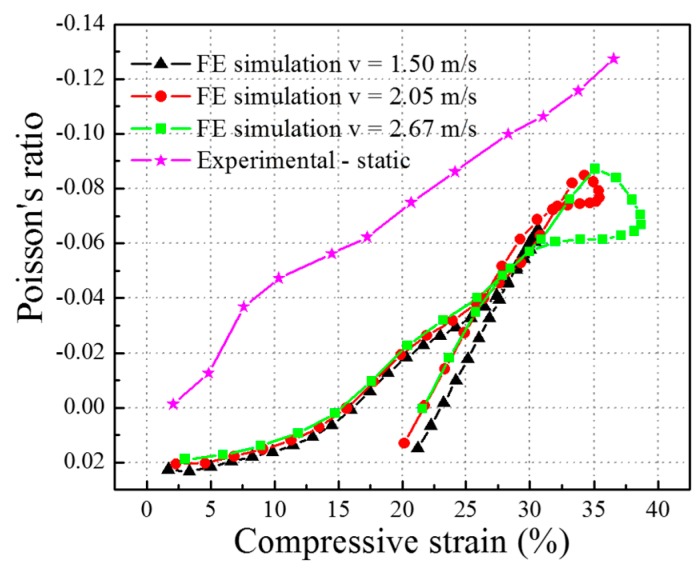
Poisson’s ratio versus compressive strain curves for the FE simulation and quasi-static compression test.

The Poisson’s ratio versus compressive strain curves of the composite at three impact velocities of 1.50 m/s, 2.05 m/s, and 2.67 m/s in the FE simulation are shown in Figure 10.

Due to the limit of the testing equipment, it is hard to obtain the real Poisson’s ratio curve of the auxetic composite from the impact experimental tests. That is also one of the most important reasons why the FE models of an auxetic composite should be established for investigating the relationship between the negative Poisson’s ratio effect and structures, as well as the constituent materials of the composites. However, the composite samples that were built in the FE analysis are the same as the samples used for static compression tests. Therefore, as one of the general mechanical properties, the Poisson’s ratio curves of the auxetic composite obtained from quasi-static compression tests could compared with those produced from impact FE modeling, to further study the influence of compression velocity on the auxetic effect.

As shown in Figure 10, it can be clearly seen that the curves under impact could be divided into two stages: the impact stage and the rebound stage. All the curves exhibit NPR after the compressive strain of 15%, but the Poisson’s ratio values are different in these two stages. After the turning point at compressive strains from 35% to 30%, the Poisson’s ratio slowly changes to a positive value, which indicates that the FE models rebound to their original states and bulge in the horizontal direction. From Figure 10, it can also be seen that the Poisson’s ratio curves under three different impact velocities highly overlap during the impact process, and only the maximum value of NPR is different. For the impact velocity of 1.50, 2.05 and 2.67 m/s, the maximum NPR value of the auxetic composite is −0.065, −0.085 and −0.087 at the compressive strain of 30.66%, 34.27% and 35.08%, respectively. Therefore, the higher the impact velocity, the larger the maximum NPR value. However, the highest NPR value for the auxetic composite under impact might not have been reached because the NPR value of the auxetic composite under the static test kept increasing after the compressive strain of 35.08%. This means that a higher NPR value than −0.087 could be obtained when the impact velocity is bigger than 2.67 m/s.

Compared with the Poisson’s ratio versus compressive strain curve of the auxetic composite obtained from the quasi-static compression test, it can be seen that the general trends of the Poisson’s ratio curves from the experimental compression and FE impact simulation are the same, but the NPR values for the quasi-static compression are bigger than those of FE impact simulation during the whole compression process. This may come from the hysteresis of the impact response. In the quasi-static compression tests, the stress wave gets fully propagated among different materials in the composite samples due to the slow increase of the compression load (2 mm/min). While under the impact tests, the stress wave could not be transmitted to the distant end of the composite in time. Therefore, this will cause the hysteresis of shrink for the sample in the horizontal direction. In other words, the NPR values of the auxetic composite under the FE impact simulation are comparatively lower than the ones obtained from static experimental testing.

### 3.3. Contact Stress Versus Compressive Strain Curves

**Figure 11:**
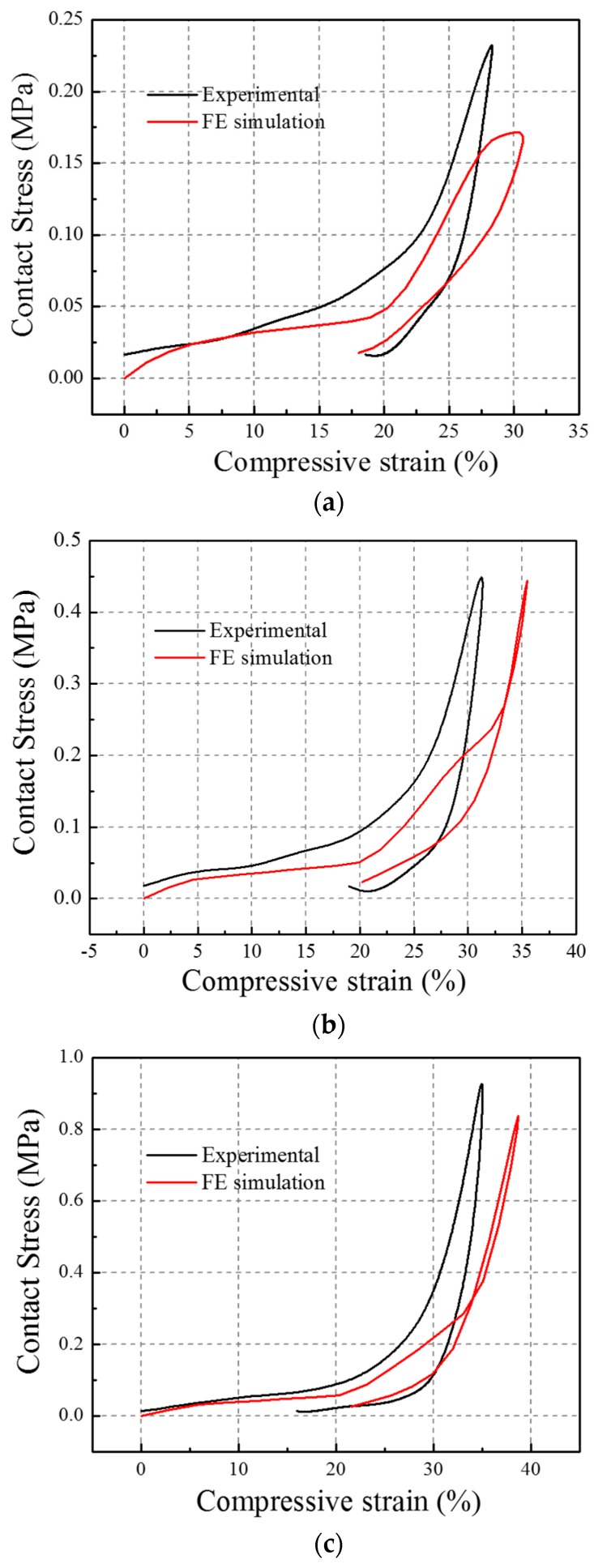
Contact stress versus compressive strain curves from the Experiment and FE simulation: (**a**) impact velocity = 1.50 m/s; (**b**) impact velocity = 2.05 m/s; (**c**) impact velocity = 2.67 m/s.

The contact stress was defined as being the contact force divided by the total cross sectional area of the composite. The FE simulation results contact stress versus compressive strain curves under three different impact velocities ranging from 1.50 m/s and 2.05 m/s to 2.67 m/s are shown in Figure 11a–c and are compared with those obtained from the experimental impact tests under the same impact velocities. The general trends of the two stress-strain curves for the FE simulation and experiment under the same impact velocity were found to be in good agreement. For example, under the impact velocity of 2.67 m/s, both curves showed a slight increase in the contact stress in the initial stage, which corresponded to a small Young’s modulus of the auxetic composite. Then, the curves reached a near-plateau region, which meant that the contact stress remained almost unchanged in the compressive strain range from 5% to 15%. Finally, the contact stress suddenly increased and roared to a high value of 0.45 MPa in the compressive strain of about 30%.

**Figure 12:**
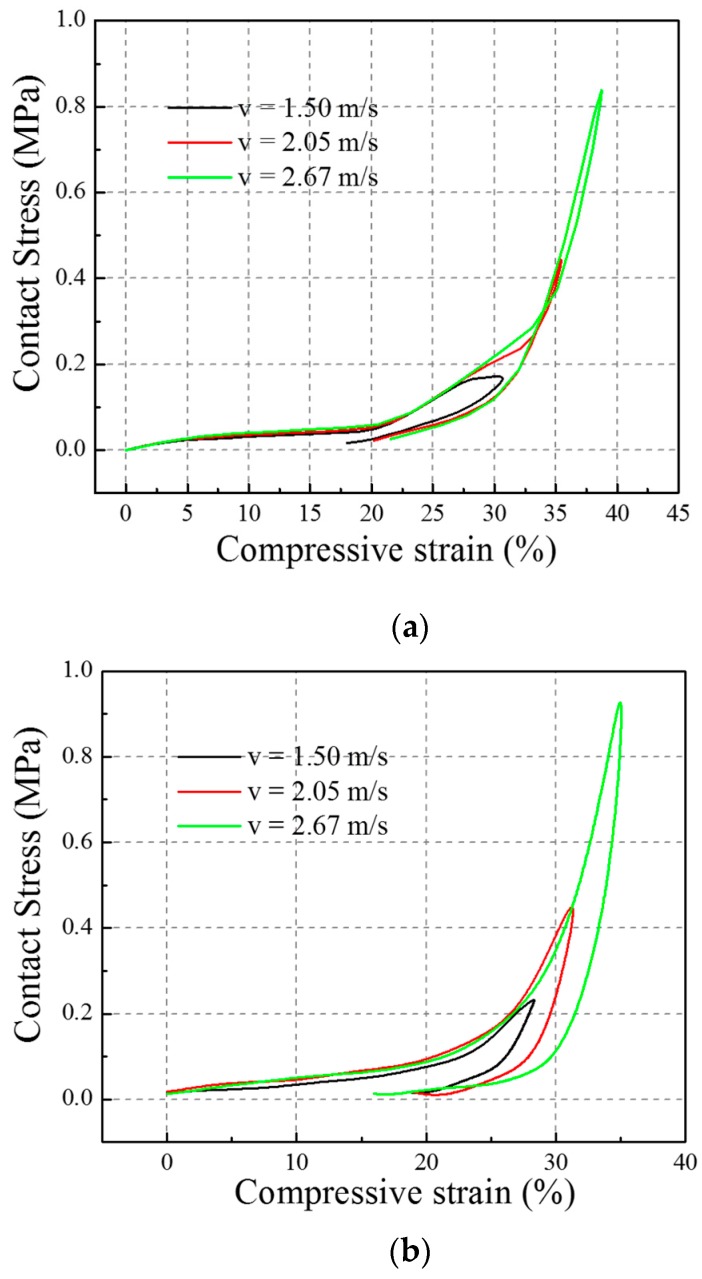
Contact stress versus compressive strain curves: (**a**) FE simulation; (**b**) Experimental.

The contact stress versus compressive strain curves under three different impact velocities in the FE simulation are shown in Figure 12a and the corresponding three contact stress-compressive strain curves under the experimental impact are shown in Figure 12b. Both figures show that the auxetic composite is impact velocity sensitive in its peak contact stress, maximum compressive strain, and initial Young’s modulus. All peak contact stress, maximum compressive strain, and initial Young’s modulus values increase with an increase in the impact velocity. A comparison between the FE simulation and experimental results in the initial Young’s modulus, the peak contact stress, and the maximum compressive strain could be found in Table 2.

The FE model for the auxetic composite that was established in the paper includes only two repeating units and the size of the FE model is far smaller than the actual sample. However, the boundary condition in the FE analysis was exactly the same with the impact testing condition. The bottom surface of the sample was fixed on the bottom plate of the testing system. The other parts of the FE model are free in the displacement of translation and rotation in all directions. Therefore, the results obtained from the numerical modelling are not precise but are representative for reference. Future work could be focused on building different sizes of FE models to investigate the effect of the number of repeating units and the repeating patterns on the mechanical response of the auxetic composite. In other words, the FE model should be further adjusted and optimized to better agree with the impact testing cases in the future.

## 4. Conclusions

In this study, a 3D FE model with two repeating units and specified boundary conditions was built in ANSYS LS-DYNA to simulate the mechanical behavior of auxetic composites under low-velocity drop-weight impact. Three different impact velocities including 1.50, 2.05 and 2.67 m/s were adopted to simulate the impact process of auxetic composites. The simulation results including the Poisson’s ratio versus compressive strain curves and the contact stress versus compressive strain curves were obtained and compared with those of experimental tests. The FE model under three different impact velocities all exhibited an obvious auxetic effect and the maximum value of the negative Poisson’s ratio occurred upon the rebounding of the composites started. Good agreement in the general trends of the stress strain curves for the FE simulation and experimental results were found. However, the corresponding values of the peak contact stress, maximum compressive strain, and initial Young’s modulus for the experimental and numerical cases are very different. This difference may come from the size effect of the FE models. Therefore, future work on building different sizes of FE models should be done to explore the effect of the number of repeating units and the repeating patterns on the mechanical response of auxetic composites.

## Figures and Tables

**Table 1:** The Specifications of Polyester Filaments and ABS Tubes.

The Polyester Filaments		The ABS Tubes	
Material density	1.38 g/cm^3^	Material density	1.05 g/cm^3^
Yarn linear density	1670 dtex (456 f)	Elastic modulus = 2.2 GPa	2.2 GPa
Elastic modulus	12.77 GPa	Bending modulus	28 GPa
Fracture stress	345.29 MPa	Poisson’s ratio	0.394
Fracture strain	4.63%	Outer Diameter	3 mm

**Table 2:** Comparison between the experimental and FE simulation results.

Impact Velocity/m/s	Initial Young’s Modulus/Mpa (Exp)	Initial Young’s Modulus/Mpa (FE)	Difference/%	Maximum Compressive Strain/% (Exp)	Maximum Compressive Strain/% (FE)	Difference/%	Peak Contact Stress/MPa (Exp)	Peak Contact Stress/MPa (FE)	Difference/%
1.50	0.21	0.34	61.90	28.33	30.68	8.30	0.23	0.17	26.09
2.05	0.39	0.55	41.03	31.32	35.45	13.19	0.45	0.44	2.22
2.67	0.41	0.58	41.46	35.05	38.70	10.41	0.93	0.84	9.68
